# Changes in Depressive Symptoms, Social Support, and Loneliness Over 1 Year After a Minimum 3-Month Videoconference Program for Older Nursing Home Residents

**DOI:** 10.2196/jmir.1678

**Published:** 2011-11-15

**Authors:** Hsiu-Hsin Tsai, Yun-Fang Tsai

**Affiliations:** ^1^School of NursingChang Gung UniversityTao-YuanTaiwan; ^2^Department of NursingChang Gung Memorial Hospital at KeelungKeelungTaiwan

**Keywords:** Videoconference, nursing home, elderly, social support, depression, loneliness

## Abstract

**Background:**

A 3-month videoconference interaction program with family members has been shown to decrease depression and loneliness in nursing home residents. However, little is known about the long-term effects on residents’ depressive symptoms, social support, and loneliness.

**Objective:**

The purpose of this longitudinal quasi-experimental study was to evaluate the long-term effectiveness of a videoconference intervention in improving nursing home residents’ social support, loneliness, and depressive status over 1 year.

**Methods:**

We purposively sampled 16 nursing homes in various areas of Taiwan. Elderly residents (N = 90) of these nursing homes meeting our inclusion criteria were divided into an experimental (n = 40) and a comparison (n = 50) group. The experimental group received at least 5 minutes/week for 3 months of videoconference interaction with their family members in addition to usual family visits, and the comparison group received regular family visits only. Data were collected in face-to face interviews on social support, loneliness, and depressive status using the Social Support Behaviors Scale, University of California Los Angeles Loneliness Scale, and Geriatric Depression Scale, respectively, at four times (baseline, 3 months, 6 months, and 12 months after baseline). Data were analyzed using the generalized estimating equation approach.

**Results:**

After the videoconferencing program, participants in the experimental group had significantly lower mean change in instrumental social support scores at 6 months (–0.42, *P* = .03) and 12 months (–0.41, *P* = .03), and higher mean change in emotional social support at 3 (0.74, *P* < .001) and 12 months (0.61, *P* = .02), and in appraisal support at 3 months (0.74, *P* = .001) after adjusting for confounding variables. Participants in the experimental group also had significantly lower mean loneliness and depressive status scores at 3 months (–5.40, *P* < .001; –2.64, *P* < .001, respectively), 6 months (–6.47, *P* < .001; –4.33, *P* < .001), and 12 months (–6.27, *P* = .001; –4.40, *P* < .001) compared with baseline than those in the comparison group.

**Conclusion:**

Our videoconference program had a long-term effect in alleviating depressive symptoms and loneliness for elderly residents in nursing homes. This intervention also improved long-term emotional social support and short-term appraisal support, and decreased residents’ instrumental social support. However, this intervention had no effect on informational social support.

## Introduction

Similar to other countries, Taiwan has more and more older people living in nursing homes. The number of nursing homes in Taiwan grew from 10 in 1995 to 372 in 2010 [1], indicating the great need for health care professionals trained in taking care of older institutionalized adults. Nursing home placement has been widely discussed in the literature as a stressful life event that challenges older people [2]. Older people who live in nursing homes have a higher prevalence of depression, which contributes to excessive morbidity [3], than do those who live in communities [4]. This prevalence of depression varied from 25% to 45% in Western countries [5,6] and was 52%–54% in Taiwan [7-9]. Many nursing home residents also experience loneliness [10], which has been associated with cognitive deterioration and hopelessness [11]. Thus, interventions have been used to enhance the quality of life in this group of people [12].

Among these interventions, social support is one of top importance because the social support systems older people use are closely related, both in quality and in quantity, to their health and quality of life [13,14]. Social support behavior falls into four categories: emotional, instrumental, informational, and appraisal support. Emotional support or affective assistance involves providing caring, empathy, love, and trust. Instrumental support refers to providing tangible goods and services or tangible aid, while informational support involves assistance with problem solving. Appraisal support or so-called affirmational support involves communicating information that is relevant to self-evaluation, rather than to problem solving [15,16].

Although most nursing home residents have become functionally dependent due to poor physical health, their psychosocial needs do not decrease [17]. In other words, social support is meaningful to them because it may provide emotional comfort. One important aspect of social support for older nursing home residents is continuous involvement of family members. However, one-third of nursing home residents were found to seldom have visitors [18]. If those older adults relocated to another nursing home, they had even fewer visitors [19]. However, support is not limited to in-person visits. Though family members may have limited time to visit residents in person, telephone calls can reduce residents’ loneliness [20]. With rapid advances in telecommunication technology, real-time audiovisual linkups are now possible among multiple locations via affordable software and hardware [21]. Providing real-time audiovisual telecommunication systems to nursing home residents in Hong Kong [21] and Japan [22] has been shown to add a new dimension for the majority who lack the skills and capacities to adapt to the nursing home environment.

The benefits of videoconferencing in medicine have been recognized as a feasible way of delivering care to frail older people living with chronic diseases [20]. Videoconferencing has also been demonstrated as a feasible way to promote social interactions among nonspeaking people living in communities [20] and between the frail elderly and their caregivers [23]. Elderly nursing home residents in Taiwan were shown by Tsai and colleagues [24] to have significantly fewer depressive symptoms and less loneliness after a 3-month program of 5 minutes/week of videoconference interactions with family members. These studies demonstrate that videoconferencing is a feasible way for individuals living either in the community or in institutions to communicate. However, those studies had small sample sizes or used a cross-sectional design. Therefore, larger longitudinal studies are needed to make causal claims about the relationship between the effectiveness of videoconferencing and participants’ depressive status and loneliness and to improve the generalizability of results.

To date, no empirical studies have used a longitudinal design to examine the effectiveness of videoconferencing for nursing home residents in Taiwan. Understanding the effectiveness of videoconferencing in Taiwanese nursing homes would fill the knowledge gap on this topic. Thus, the purpose of this study was to evaluate the long-term follow-up effectiveness of a videoconference intervention program on nursing home residents’ social support, loneliness, and depressive status after a minimum 3-month videoconference program.

## Methods

### Design, Sample, and Setting

A quasi-experimental longitudinal design was used. Nursing homes from northern and central Taiwan were purposively selected based on three criteria: size (>65 beds), with Internet access, and accessible to the researchers. Among 23 medium-large nursing homes that met the recruitment criteria, 7 declined to participate in our study. The remaining 16 nursing homes (total beds =2190) were therefore randomly assigned to the comparison or experimental group.

The sample size was estimated based on the rule that 15 participants were needed for each variable [25]. Since we tested three major variables (depression, loneliness, and social support), the ideal sample size for this study was 45. Residents in the 16 nursing homes were recruited if they met the following criteria: (1) older than 60 years, (2) Mini-Mental State Examination (MMSE) score ≥16 for participants with no formal education or MMSE score >20 for those with at least a primary school education [26,27], and (3) wireless Internet access on their residence floor. Residents’ family members had to have access to the Internet and be familiar with Internet communication programs such as Skype. These inclusion criteria were met by 423 residents, who were invited along with their family members to participate in the study. Family members of the majority of residents (n = 333, 78.7%) declined to participate, resulting in 50 participants in the comparison group and 40 in the experimental group. Residents in the experimental group used videoconferencing to communicate with their families plus their usual communication activities, whereas residents in the comparison group maintained their usual activities.

### Videoconference Program

The videoconference program used laptops and Internet communication programs. Nursing home residents were asked to use the Internet at least once a week, with help from a trained research assistant, who spent at least 5 minutes per week with each resident for the first 3 months during their scheduled videoconference visit. This weekly frequency was chosen to reflect the frequency of in-person visits to a nursing home resident for the majority of families [28,29], and 3 months was chosen to allow them time to adjust to the new videoconference program [30,31]. After 3 months, whenever residents wanted to have a video communication with their family, they were helped by the nursing home staff (nurses or nurses’ aides) who were trained by the authors. The residents’ main family contact person was a spouse, child, grandchild, or significant other. The communication programs used were Windows Live Messenger (MSN; Microsoft Corporation, Redmond, WA, USA) or Skype (Skype Technologies SA, Luxembourg) via a 2 M/256 K wireless modem run on a large-screen (15.6 inch) laptop.

### Procedure

The study was approved by the Institutional Review Board of the authors’ institution. After permission was granted from the nursing homes’ administrators, details of our research procedure were posted at the entrance of each nursing home. This announcement indicated that residents or family members interested in participating in the study could directly contact the study personnel or nursing home staff. We also asked nursing home staff to talk with residents who met our study criteria and their family members about their willingness to participate in this study. Those who were interested in participation were contacted by the research assistant, who explained their rights, benefits, confidentiality, and responsibilities when participating in the study. After signing informed consent, residents and family members made appointments for videoconferences. Family members were phoned or emailed before the scheduled time to remind them of the appointment. The family of residents in the experimental group could continue their in-person or telephone visits as usual. Laptops were left in the nursing homes for 1 year. For the first 3 months, residents were helped by a trained research assistant to use the videoconference technology in a private room; for the next 9 months, residents were helped by trained nursing home staff. All residents in both the experimental and comparison groups completed questionnaires for demographic information (baseline only), depressive symptoms, loneliness, and social support at baseline and at 3, 6, 9, and 12 months.

### Study Variables

Demographic indicators included participants’ age, gender, marital status, educational background, duration of residency in the nursing home, and frequency of family visits. Residents’ physical status and cognitive status were measured at baseline using the Barthel Index [32], which assesses performance of activities of daily living (ADLs), and MMSE [26], respectively. The MMSE cut-off score for severe cognitive deficit is ≥16 for participants without formal education and ³20 for those with at least a primary school education [27].

Depressive status was measured by the Geriatric Depression Scale (GDS) [33]. The GDS has 30 items with a yes/no response set. Possible scores range from 0 to 30. The GDS cut-off score for depressive symptom is >10 for mild depression and >20 for severe depression. The reliability of the GDS in a previous study of Taiwanese nursing home elderly was 0.91 [24] and in this study was 0.84.

Loneliness was measured by the revised University of California Los Angeles (UCLA) Loneliness Scale [34]. Responses to the 10 items on the UCLA Loneliness Scale are rated on a 4-point Likert scale from 1 (never) to 4 (always). Possible scores range from 20 to 80, with higher scores indicating higher perceived loneliness. The reliability of the UCLA Loneliness Scale was 0.87 in a study of institutionalized elderly in Taiwan [35] and was 0.92 in the current study.

Social support was measured by the Social Support Behaviors Scale with three subscales: social support network, quantity of social support, and satisfaction with social support [36]. Social support network was measured by the number of family members or friends who contacted residents and the number of contacts (either by phone or in person) during the previous week. The quantity of social support was measured by asking participants to rate each social support behavior (emotional, informational, instrumental, and appraisal support) offered by different providers (spouse, children, relatives, neighbors, and friends). Responses to this 14-item subscale are rated on a 5-point Likert scale, with higher scores indicating more support from each social resource. The subscale reliabilities for social support network, quantity of social support, and satisfaction with social support were 0.71, 0.92, and 0.77, respectively in Taiwanese nursing home residents [24], and 0.72, 0.89, and 0.79, respectively, in this study.

Family involvement with residents was confirmed by asking nursing home staff to record the number of visits and phone calls made to the residents. The duration of each videoconference interaction during the year was recorded by either the research assistant or nursing staff. Videoconference use was coded as the frequency of all videoconference interactions per month.

### Data Analysis

All data were coded before being entered into a computer. Statistical analyses were performed using SPSS for Windows version 15.0 (IBM Corporation, Somers, NY, USA). Participants’ demographic data were analyzed by descriptive statistics. Differences between groups were compared at four points (baseline, 3 months, 6 months, and 12 months) using multiple linear regression with the generalized estimating equation (GEE) method [37]. That is, we used the GEE method’s multiple linear regression model (with the time and group interaction) to compare the time effects between two groups with or without adjusting for the effects of confounding variables such as resident’s age and length of nursing home residency. Statistical significance was defined as *P* < .05.

## Results

### Participants’ Characteristics

The 40 participants in the experimental group were on average 73.82 (SD 11.19) years old at baseline. The experimental group’s use of videoconferencing decreased over time at 3, 6, and 12 months: mean (SD) 2.09 (1.46), 1.69 (1.37), and 1.14 (1.22), respectively. However, this decrease was not statistically significant. The majority of participants were female (22/40, 55%) and widowed (29/40, 73%), and 35% (14/40) had graduated from primary school. Their average MMSE and Barthel Index scores at baseline were 23.51 (SD 3.93) and 65.68 (SD 22.55), respectively, indicating good cognitive status and above average performance of ADLs. They had an average of 3.69 (SD 2.09) children. About half of these participants (22/40, 55%) were visited by a family member at least once a week, and only 18% (7/40) seldom (less than once a month) had a family member visit them. Their average length of residency was 28.38 (SD 30.79) months (Table 1).

**Table 1:** Demographic characteristics of experimental and comparison groups

Variable		Comparison group (n = 50)		Experimental group (n = 40)		χ^2^(df^a^; *P* value)	*t*(*P* value) df^a^ = 88
		n (%)	Mean (SD)	n (%)	Mean (SD)		
Age (years)			79.26 (7.07)		73.82 (11.19)		10.78 (.01)
**Gender**						0.2 (1; .63)	
	Male	20 (40)		18 (45)			
	Female	30 (60)		22 (55)			
**Marital status**						6.6 (3; .16)	
	Single	1 (2)		2 (5)			
	Married	20 (40)		8 (20)			
	Divorced	3 (6)		1 (2)			
	Widowed	26 (52)		29 (73)			
**Education**						12.7 (4; .06)	
	None/illiterate	29 (58)		9 (23)			
	Primary	8 (16)		14 (35)			
	Junior high school	2 (4)		2 (5)			
	Senior high school	8 (16)		11 (28)			
	≥ College	3 (6)		4 (10)			
Number of children			3.90 (2.05)		3.69 (2.09)		0.10 (.64)
Residency (months)			29.32 (28.58)		28.38 (30.79)		0.04 (.87)
Barthel Index			63.10 (23.62)		65.68 (22.55)		0.16 (.61)
MMSE			22.22 (3.93)		23.51 (3.93)		0.05 (.13)
**In-person visits**							
	None/seldom	7 (14)		7 (18)			
	Monthly	5 (10)		11 (28)			
	Weekly (>2/month)	34 (68)		18 (45)			
	Daily (>5/week)	4 (8)		4 (10)			
**Telephone calls (number/week)**							
	0	36 (72)		24 (60)			
	1	10 (20)		8 (20)			
	2–6	3 (6)		6 (15)			
	≥7	1 (2)		2 (5)			

^a^ Degrees of freedom.

The 50 participants in the comparison group were on average 79.26 (SD 7.07) years old at baseline. The majority were female (30/50, 60%), had no formal education (29/50, 58%), and were widowed (26/50, 52%). Their average MMSE and Barthel Index scores were 22.22 (SD 3.93) and 63.10 (SD 23.62), respectively, indicating good cognitive status and above average performance of ADLs. Their average number of children was 3.90 (SD 2.05). Most participants (38/50, 76%) were visited by a family member at least once a week and 14% (7/50) seldom had a family member visit them. Their average length of residency was 29.32 (SD 28.58) months (Table 1). The experimental and comparison group did not differ significantly in any demographic characteristics except for age (*t* = 10.78, *P* = .01).

**Figure 1:**
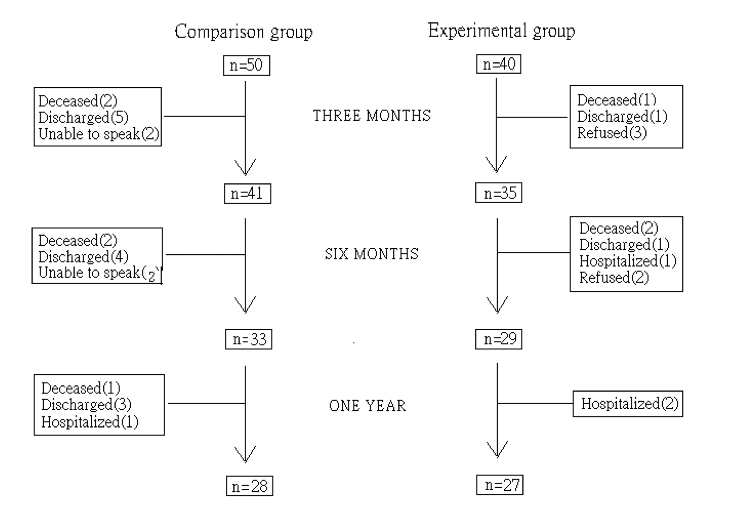
Attrition of participants in the two groups over the 1-year study period.

During the 12-month study, 13 participants in the experimental group withdrew from the study (including 5 who declined to continue participating, 2 who relocated back home, and 3 who died), with an attrition rate of 33%. In the comparison group, 22 participants dropped out (including 5 who died, 12 who relocated back home, and 4 who developed cognitive deficits), with an attrition rate of 44% (Figure 1). Participants who dropped out and those who remained in the study did not differ significantly in any demographic characteristics.

### Outcomes

Descriptive analysis of outcomes shows that both groups had the highest social support scores for the informational and instrumental social support subscales. The mean GDS scores for depressive status at baseline, and 3, 6, and 12 months were 12.75, 11.57, 12.85, and 13.00, respectively, for the experimental group, and 10.52, 10.56, 14.41, and 15.04, respectively, for the comparison group. These GDS scores did not differ significantly by independent *t* test between the two groups at any time (Table 2). Mean UCLA loneliness scores at baseline, and 3, 6, and 12 months were 49.70, 44.54, 46.21, and 45.92, respectively, for the experimental group, and 45.76, 45.59, 47.81, and 48.32, respectively, for the comparison group. The mean loneliness scores did not differ significantly between the two groups at any time (Table 2).

**Table 2:** Social support (Social Support Behaviors Scale), depressive status (Geriatric Depression Scale), and loneliness (University of California Los Angeles Loneliness Scale) scores by group at baseline, and 3, 6, and 12 months

Variable		Comparison group (n = 50)		Experimental group (n = 40)		*t* (*P* value) df^a^ = 88
		Mean	SD	Mean	SD	
**Social support: emotional support**						
	Baseline	9.76	1.69	9.49	1.57	0.78 (.59)
	3 months	9.40	1.55	9.71	1.72	–0.80 (.66)
	6 months	9.24	1.28	9.30	1.67	–0.14 (.24)
	12 months	8.96	0.98	9.31	1.80	–2.30 (.04)
**Social support: informational support**						
	Baseline	10.66	1.82	11.13	1.48	–1.31 (.21)
	3 months	10.62	1.20	11.31	1.41	–2.33 (.02)
	6 months	10.60	1.55	11.02	1.43	–1.08 (.61)
	12 months	10.28	1.32	10.76	1.63	–1.16 (.25)
**Social support: instrumental support**						
	Baseline	10.40	1.05	10.50	1.22	–0.38 (.33)
	3 months	10.36	0.84	10.35	1.26	0.05 (.95)
	6 months	10.53	1.05	10.31	1.14	0.77 (.51)
	12 months	10.13	0.83	10.01	1.17	0.45 (.12)
**Social support: appraisal support**						
	Baseline	9.25	1.60	9.03	1.25	0.73 (.14)
	3 months	8.83	1.26	9.30	1.32	–1.59 (.12)
	6 months	8.98	1.28	9.30	1.58	–0.87 (.39)
	12 months	8.70	1.22	9.26	1.67	–1.42 (.16)
**Total social support**						
	Baseline	141.54	18.64	141.85	17.26	–0.08 (.93)
	3 months	139.00	14.95	143.11	18.36	–1.07 (.28)
	6 months	139.55	16.08	140.43	18.02	–0.20 (.63)
	12 months	134.08	12.74	138.08	19.36	–0.85 (.40)
**Depressive status**						
	Baseline	10.52	4.06	12.75	5.50	1.94 (.06)
	3 months	10.56	3.89	11.57	5.27	–0.96 (.34)
	6 months	14.41	4.93	12.85	5.35	1.16 (.25)
	12 months	15.04	4.61	13.00	4.50	1.60 (.11)
**Loneliness**						
	Baseline	45.76	9.73	49.70	10.25	–1.85 (.09)
	3 months	45.59	9.40	44.54	12.68	0.41 (.68)
	6 months	47.81	9.97	46.21	11.87	0.57 (.56)
	12 months	48.32	10.17	45.92	12.14	0.78 (.47)

^a^ Degrees of freedom.

Time effects between the two groups were compared using the GEE method’s multiple linear regression. As shown in Table 3, the average UCLA Loneliness Scale score of the experimental group was higher at baseline than that of the comparison group (beta = 3.94, *P* = .09). On the other hand, the changes in UCLA Loneliness Scale scores for the experimental group at 3, 6, and 12 months (compared with baseline) were significantly lower (–4.84, –6.46, and –6.42, respectively, all *P* < .001) than the corresponding changes in loneliness scores for the comparison group (–0.31, 2.81, and 2.77, respectively, with corresponding *P* values = .55, .02, and .02) (Table 3). Moreover, after adjusting for the effects of residents’ age and length of residency, all aforementioned results remained almost the same (right side of Table 3). Similarly, after controlling for residents’ age and length of residency, the changes in GDS scores were on average significantly lower in the experimental group than in the comparison group at 3, 6, or 12 months (beta = –2.64, –4.33, and –4.40, respectively, all *P* < .001).

**Table 3:** Effects of videoconference intervention on participants’ depressive status and loneliness at 3, 6, and 12 months in consideration of group, time, and time × group effects

Variable			Unadjusted				Adjusted^a^			
			beta	SE	χ^2^_1_	*P* value	beta	SE	χ^2^_1_	*P* value
**Depressive status**										
	**Group**									
		E vs C^b^	2.22	1.03	5.0	.06	2.26	1.00		5.1	.05
	** Time (vs B^c^) **									
		3 months	0.97	0.33	8.9	<.001	0.99	0.32	9.3	<.001
		6 months	6.95	0.63	123.4	<.001	6.99	0.63	142.7	<.001
		12 months	7.64	0.70	118.9	<.001	7.71	0.70	119.6	<.001
**Time × group^d^**										
		3 months	–1.36	0.56	6.0	.02	–2.64	0.57		21.3	<.001
		6 months	–4.50	0.97	21.6	<.001	–4.33	1.03		17.6	<.001
		12 months	–4.45	0.89	24.9	<.001	–4.40	0.92		23.1	<.001
**Loneliness**										
	**Group**									
		E vs C^b^	3.94	2.10	3.5	.09	3.27	2.23	2.2	.14
	**Time (vs B^c^)**									
		3 months	–0.31	0.53	0.4	.55	–0.31	0.53	0.3		.56
		6 months	2.81	1.23	5.2	.02	2.81	1.23	5.2		.02
		12 months	2.77	1.22	5.2	.02	2.78	1.23	5.1		.02
**Time × group^d^**										
		3 months	–4.84	1.14	18.0	<.001	–5.40	1.22	19.6	<.001
		6 months	–6.46	1.64	15.4	<.001	–6.47	1.70	14.5	<.001
		12 months	–6.42	1.64	15.3	<.001	–6.27	1.94	10.5	.001

^a^ Adjusted for residents’ age and length of residency.

^b^ C: comparison group, E: experimental group.

^c^ B: baseline measurement.

^d^ Group 0: comparison group (reference group), group 1: experimental group.

This study had nine outcome variables of interest (depressive status, loneliness, total social support, emotional support, informational support, instrumental support, appraisal support, number of phone calls, and number of visits). Each outcome variable was analyzed exactly as in Table 3, but for each variable, we were mainly interested in comparing the time effects between the experimental and comparison groups. For simplicity, changes in only the time and group interaction terms are summarized in Table 4. The changes in appraisal and emotional social support scores at 3 months, after adjusting for the effects of residents’ age and length of residency, were on average significantly higher in the experimental group than the corresponding changes in the comparison group (both beta = 0.74, *P*. = 001 and *P* < .001). The changes in instrumental social support scores at 6 and 12 months were on average significantly lower in the experimental group than in the comparison group (beta = –0.42 and –0.41, respectively, both *P* = .03) after adjusting for the effects of residents’ age and length of residency (Table 4).

**Table 4:** Effects of videoconference intervention on participants’ social support, depressive status, and loneliness at 3, 6, and 12 months in consideration of time × group effects

Variable		Unadjusted				Adjusted^a^			
		beta	SE	χ^2^_1_	*P* value	beta	SE	χ^2^_1_	*p* value
**Total social support (time × group^b^)**									
	3 months	3.71	2.10	3.1	.08	3.63	2.10	3.0	.09
	6 months	–0.58	2.50	0.1	.82	–0.84	2.52	0.1	.74
	12 months	–0.05	2.73	0.0	.99	–0.48	2.74	0.0	.86
**Emotional support (time × group ^b^)**									
	3 months	0.60	0.19	10.1	.001	0.74	0.19	15.3	<.001
	6 months	0.32	0.26	1.5	.23	0.40	0.28	2.1	.15
	12 months	0.47	0.25	3.5	.06	0.61	0.26	5.3	.02
**Informational support (time × group ^b^)**									
	3 months	–0.00	0.23	0.0	>.99	0.15	0.25	0.4	.53
	6 months	–0.29	0.27	1.2	.28	–0.24	0.31	0.6	.44
	12 months	–0.34	0.31	1.2	.28	–0.18	0.36	0.3	.62
**Instrumental support (time × group ^b^)**									
	3 months	–0.20	0.14	2.0	.16	–0.14	0.15	0.9	.34
	6 months	–0.47	0.18	6.7	.01	–0.42	0.19	4.7	.03
	12 months	–0.41	0.19	4.9	.03	–0.41	0.19	4.6	.03
**Appraisal support (time × group ^b^)**									
	3 months	0.66	0.22	9.3	.002	0.74	0.22	10.8	.001
	6 months	0.37	0.24	2.3	.13	0.43	0.26	2.8	.10
	12 months	0.57	0.31	3.5	.06	0.58	0.32	3.3	.07
**Depressive status (time × group ^b^)**									
	3 months	–1.36	0.56	6.0	.02	–2.64	0.57	21.3	<.001
	6 months	–4.50	0.97	21.6	<.001	–4.33	1.03	17.6	<.001
	12 months	–4.45	0.89	24.9	<.001	–4.40	0.92	23.1	<.001
**Loneliness (time × group ^b^)**										
	3 months	–4.84	1.14	18.0	<.001	–5.40	1.22	19.6	<.001
	6 months	–6.46	1.64	15.4	<.001	–6.47	1.70	14.5	<.001
	12 months	–6.42	1.64	15.3	<.001	–6.27	1.94	10.5	.001
**Number of telephone calls (time × group ^b^)**									
	3 months	0.28	0.11	6.3	.01	0.28	0.12	5.8	.02
	6 months	0.22	0.14	2.5	.11	0.20	0.15	1.7	.19
	12 months	–0.01	0.15	0.0	.97	0.01	0.16	0.0	.95
**Number of visits (time × group ^b^)**										
	3 months	–0.08	0.07	1.6	.21	–0.08	0.07	1.2	.27
	6 months	0.05	0.08	0.4	.53	0.03	0.08	0.2	.70
	12 months	0.05	0.08	0.4	.55	0.04	0.08	0.2	.67

^a^ Adjusted for residents’ age and length of residency.

^b^ Group 0: comparison group (reference group), group 1: experimental group.

### Discussion

This study demonstrated that our videoconference intervention alleviated elderly nursing home residents’ perceived loneliness and improved their depressive status at 3, 6, and 12 months after the intervention. However, instrumental social support decreased at 6 and 12 months after the intervention.

Our 1-year attrition rate was high (35/90, 39%), as previously reported in similar longitudinal studies [38,39]. For example, an attrition rate of 39.5% was reported in a study on the effects of Internet use on health and depression [39]. Our attrition rate at 6 months (28/90, 31%) was also similar to the 6-month attrition rate (22.90%) reported for a study of elderly nursing home residents in Taiwan [38]. Among the reasons for case loss in our study, 16% (14/90) were discharged home, close to 15.26% as previously reported [38]. The majority of discharges were because the residents’ families could not pay for the nursing home during the data collection period, which coincided with an economic depression in Taiwan. Other residents were discharged home because they were healthier than at admission.

Our research found that videoconferencing effectively improved elderly residents’ depressive status at 3, 6, and 12 months. These results are consistent with a previous report [39] that using the Internet for communication with friends and family was associated with small but reliable decreases in depression. However, our study results are different from another report [40] of no significant difference in depression and loneliness among older adults after 5 months of training to access the Internet and email. In that study, however, participants were only trained to access the Internet, not to specifically contact family members or significant others [40]. The results of a previous study of institutionalized older Chinese adults [8] indicate that only family members can comfort these residents and reduce their depression and loneliness. This finding likely explains why depression and loneliness did not significantly change after intervention in White and colleagues’ study [40]. In our program, not only were elderly residents shown how to use the Internet, but also appointments were arranged for them to communicate with their family members, who provide the majority of social support to Chinese elders and therefore reduce their depressive symptoms [8].

We found that videoconferencing effectively improved elderly residents’ loneliness at 3, 6, and 12 months. These results are consistent with those of another study done in the United States [41] showing that loneliness was significantly reduced in 22 community-dwelling elderly people after 4 months of computer use. These results might be due to videoconference use providing a “social presence” to older adults [42]. For elderly residents in nursing homes, videoconferencing might add color to their lives. These results suggest that videoconference use is a good way to reduce loneliness of the elderly in both the community and institutions.

Our research found that videoconferencing, a computer-mediated communication, had no effects on instrumental social support at the 3-month data collection time, as previously reported [43,44]. However, we found that instrumental social support decreased significantly over time, but not the number of family members’ in-person visits. In other words, family members provided less instrumental social support in terms of specific items and assistance, even though they kept visiting the elderly residents in person. After a long stay in a nursing home, elderly residents tend to adapt to the environment and not need extra items for daily life, since such things are provided by the nursing home. Thus, family members might not see instrumental social support as the best way to show their filial piety, or the elders might not ask family members to bring them things. These possibilities need to be examined in future studies.

Our videoconference intervention also improved emotional social support at 3 and 12 months and appraisal support after 3 months. The effect of videoconferencing on appraisal and emotional support at 3 months is similar to our previous study [24]. The lack of intervention effects on appraisal social support at 6 and 12 months might be due to decreased novelty and quality of videoconferencing. Although videoconferencing is a convenient way to connect with people at a distance, videoconferencing alone cannot improve the quality and amount of communications between people. In particular, when communicators view the communication as an obligation, they might feel bored, shorten the communication, or show an unpleasant attitude or tone.

From this point of view, we suggest that nursing home administrators increase the quality of communication by developing an interaction program such as arranging for family members to have a meal with residents at the nursing home and have a meal together via videoconference. One explanation for the long-term (12 months) decrease in emotional social support might be that nursing home residents feel safe or comforted by using videoconferencing as an alternative “social presence” so that they can immediately see their family member, even at a distance. Videoconferencing may offer them a chance to be part of family life. They also might feel comforted by seeing their family member’s actual state and would not worry that the family member was hiding a problem to allay anxiety if he or she could not visit [45].

The use of videoconference visits decreased over time. This decreased use of videoconferencing was likely due to a loss in the novelty of videoconferencing, lack of staff to help the residents operate the devices, and a need to remind family members to use videoconferencing (busy family members tended to forget to use videoconferencing). However, these possible reasons for decreased videoconference use need to be supported by further research. Furthermore, we found that videoconference use was high in some nursing homes, especially for those residents with relatives living overseas. However, our data were not significant due to the small sample size from each nursing home. We found no studies on the relationships between videoconference use and the characteristics of nursing home residents’ families. We therefore suggest that future research explore the relationships between videoconference use and characteristics of nursing home residents’ family members, factors influencing videoconference use, effects of videoconferencing on the health of elderly residents and their families, and the cost effectiveness of the videoconference program.

Although the experimental and control groups did not differ significantly in any dependent variable at any time point, the experimental group showed significant changes in depression, loneliness, and two social support measures over time compared with the control group after controlling for residents’ age and length of residency. The independent *t* test used to compare the results for each outcome variable at each measurement time point (Table 2) did not benefit from the strength of repeated measurements within participants. One possible reason that the independent *t* test did not show significant differences in mean outcome measurements between groups is that it did not include other repeated measurements from the same participants. Another possible reason is that the sample was too small to detect significant differences. On the other hand, the results of GEE analysis showed significant incremental changes in the dependent variables over time due to the likely impact of within-participant’s repeated measurements.

Our research also showed that, after adjustment for residents’ age and length of residency, the time effects between the experimental and comparison groups remained the same for all outcome variables except emotional social support at 12 months. In other words, after adjustment for time and group effects, age and length of residency had almost no significant impact on all outcome variables, except emotional social support at 12 months.

Furthermore, the outcome variables of loneliness, lack of social support, and depression might have been associated with each other. However, when we analyzed each variable by GEE method with and without controlling for other variables, we found that the trends were not affected (data not shown). Further research is recommended to explore the associations among these variables and their possible impact on the time effects.

#### Conclusion
